# Correntropy Based Matrix Completion

**DOI:** 10.3390/e20030171

**Published:** 2018-03-06

**Authors:** Yuning Yang, Yunlong Feng, Johan A. K. Suykens

**Affiliations:** 1 College of Mathematics and Information Science, Guangxi University, Nanning 530004, China; 2 Department of Mathematics and Statistics, The State University of New York at Albany, Albany, NY 12222, USA; 3 Department of Electrical Engineering, ESAT-STADIUS, KU Leuven, Kasteelpark Arenberg 10, Leuven B-3001, Belgium

**Keywords:** robust matrix completion, hard/soft iterative thresholding, non-Gaussian noise, outliers, linear convergence

## Abstract

This paper studies the matrix completion problems when the entries are contaminated by non-Gaussian noise or outliers. The proposed approach employs a nonconvex loss function induced by the maximum correntropy criterion. With the help of this loss function, we develop a rank constrained, as well as a nuclear norm regularized model, which is resistant to non-Gaussian noise and outliers. However, its non-convexity also leads to certain difficulties. To tackle this problem, we use the simple iterative soft and hard thresholding strategies. We show that when extending to the general affine rank minimization problems, under proper conditions, certain recoverability results can be obtained for the proposed algorithms. Numerical experiments indicate the improved performance of our proposed approach.

## 1. Introduction

Arising from a variety of applications such as online recommendation systems [1,2], image inpainting [3,4] and video denoising [5], the matrix completion problem has drawn tremendous and continuous attention over recent years [6,7,8,9,10,11,12]. The matrix completion aims at recovering a low rank matrix from partial observations of its entries [7]. The problem can be mathematically formulated as:

(1) $$\min_{X\in R^{m\times n}}\;\;\mathrm{rank}\left(X\right)\;\;\;s.t.\;\;\;X_{ij}=B_{ij},\;\;\left(i,j\right)\in \Omega ,$$

where $X,\;B\in R^{m\times n}$ and Ω is an index set. Due to the nonconvexity of the rank function rank(·), solving this minimization problem is NP-hard in general. To obtain a tractable convex relaxation, the nuclear norm heuristic was proposed [7]. Incorporated with the least squares loss, the nuclear norm regularization was proposed to solve (1) when the observed entries are contaminated by Gaussian noise [13,14,15,16]. In real-world applications, datasets might be contaminated by non-Gaussian noise or sparse gross errors, which can appear in both explanatory and response variables. However, it has been well understood that the least squares loss cannot be resistant to non-Gaussian noise or outliers.

To address this problem, some efforts have been made in the literature. Ref. [17] proposed a robust approach by using the least absolute deviation loss. Huber’s criterion was adopted in [18] to introduce robustness into matrix completion. Ref. [19] proposed to use an $L_{p}$ (0<p≤1) loss to enhance the robustness. However, as explained later, the approaches mentioned above cannot be robust to impulsive errors. In this study, we propose to use the correntropy-induced loss function in matrix completion problems when pursuing robustness.

Correntropy, which serves as a similarity measurement between two random variables, was proposed in [20] within the information-theoretic learning framework developed in [21]. It is shown that in prediction problems, error correntropy is closely related to the error entropy [21]. The correntropy and the induced error criterion have been drawing a great deal of attention in the signal processing and machine learning community. Given two scalar random variables U, V, the correntropy $V_{\sigma }$ between *U* and *V* is defined as $V_{\sigma }\left(U,V\right)=EK_{\sigma }\left(U,V\right)$ with $K_{\sigma }$ a Gaussian kernel given by $K_{\sigma }\left(u,v\right)=\exp\left\{-{\left(u-v\right)}^{2}/\sigma^{2}\right\}$, the scale parameter σ>0 and (u,v) a realization of (U,V). It is noticed in [20] that the correntropy $V_{\sigma }\left(U,V\right)$ can induce a new metric between *U* and *V*.

In this study, by employing the correntropy-induced losses, we propose a nonconvex relaxation approach to robust matrix completion. Specifically, we develop two models: one with a rank constraint and the other with a nuclear norm regularization term. To solve them, we propose to use simple, but efficient algorithms. Experiments on synthetic, as well as real data are implemented and show that our methods are effective even for heavily-contaminated datasets. We make the following contributions in this paper:

- In Section 3, we propose a nonconvex relaxation strategy for the robust matrix completion problem, where the robustness benefits from using a robust loss. Based on this loss, a rank constraint, as well as a nuclear norm penalized model is proposed. We also extend the proposed models to deal with the affine rank minimization problem, which includes the matrix completion as a special case.
- In Section 4, we propose to use simple, but effective algorithms to solve the proposed models, which are based on gradient descent and employ the hard/soft shrinkage operators. By verifying the Lipschitz continuity, the convergence of the algorithms can be proven. When extended to affine rank minimization problems, under proper conditions, certain recoverability results are obtained. These results give understandings of this loss function in an algorithmic sense, which is in accordance with and extends our previous work [22].

This paper is organized as follows: In Section 2, we review some existing (robust) matrix completion approaches. In Section 3, we propose our nonconvex relaxation approach. Two algorithms are proposed in Section 4 to solve the proposed models. Theoretical results will be presented in Section 4.1. Experimental results are reported in Section 5. We end this paper in Section 6 with concluding remarks.

## 2. Related Work and Discussions

In matrix completion, solving the optimization problem in Model (1) is NP-hard, and a usual remedy is to consider the following nuclear norm convex relaxation:

(2) $$\min_{X\in R^{m\times n}}\;\;{∥X∥}_{*}\;\;\;\;s.t.\;\;\;X_{i,j}=B_{i,j},\;\;\left(i,j\right)\in \Omega .$$

Theoretically, it has been demonstrated in [7,8] that under proper assumptions, with an overwhelming probability, one can reconstruct the original matrix. Situations of the matrix completion with noisy entries have been also considered; see, e.g., [6,9]. In the noisy setting, the corresponding observed matrix turns out to be:

(3) $$\begin{array}{c} B_{\Omega }=X_{\Omega }+E, \end{array}$$

where $B_{\Omega }$ denotes the projection of *B* onto Ω, and *E* refers to the noise. The following two models are frequently adopted to deal with the noisy case:

$$\begin{array}{c} \min_{X\in R^{m\times n}}\;\;\frac{1}{2}{∥X_{\Omega }-B_{\Omega }∥}_{F}^{2}\;\;\;\;\;\;s.t.\;\;\mathrm{rank}\left(X\right)\leq R, \end{array}$$

and its convex relaxed and regularized heuristic:

$$\begin{array}{c} \min_{X\in R^{m\times n}}\;\;\frac{1}{2}∥X_{\Omega }-B_{\Omega }{∥}_{F}^{2}+\lambda {∥X∥}_{*}, \end{array}$$

where λ>0 is a regularization parameter. Similar theoretical reconstruction results have been also derived in the noiseless case under technical assumptions. Along this line, various approaches have been proposed [14,15,16,23,24]. Among others, Refs. [10,25] interpreted the matrix completion problem as a specific case of the trace regression problem endowed with an entry-wise least squares loss, ${∥\cdot ∥}_{F}^{2}$. In the above-mentioned settings, the noise term *E* is usually assumed to be Gaussian or sub-Gaussian to ensure the good generalization ability, which certainly excludes the heavily-tailed noise and/or outliers.

### Existing Robust Matrix Completion Approaches

It has been well understood that the least squares estimator cannot deal with non-Gaussian noise or outliers. To alleviate this limitation, some efforts have been made.

In a seminal work, Ref. [17] proposed a robust matrix completion approach, in which the model takes the following form:

(4) $$\min_{X,E\in R^{m\times n}}{\;\;∥E∥}_{1}+\lambda {∥X∥}_{*}\;\;\;\;s.t.\;\;X_{\Omega }+E=B_{\Omega }.$$

The above model can be further formulated as:

$$\min_{X\in R^{m\times n}}\;\;∥X_{\Omega }-B_{\Omega }{∥}_{1}+\lambda {∥X∥}_{*},$$

where λ>0 is a regularization parameter. The robustness of the model (4) results from using the least absolute deviation loss (LAD). This model was later applied to the column-wise robust matrix completion problem in [26].

By further decomposing *E* into $E=E_{1}+E_{2}$, where $E_{1}$ refers to the noise and $E_{2}$ stands for the outliers, Ref. [18] proposed the following robust reconstruction model:

$$\min_{X,E_{2}\in R^{m\times n}}\;\;∥X_{\Omega }-B_{\Omega }-E_{2}{∥}_{F}^{2}+{\lambda ∥X∥}_{*}+\gamma {∥E_{2}∥}_{1},$$

where λ,γ>0 are regularization parameters. They further showed that the above estimator is equivalent to the one obtained by using Huber’s criterion when evaluating the data-fitting risk. We also note that [19] adopted an $L_{p}$ (0<p≤1) loss to enhance the robustness.

## 3. The Proposed Approach

### 3.1. Our Proposed Nonconvex Relaxation Approach

As stated previously, matrix completion models based on the least squares loss cannot perform well with non-Gaussian noise and/or outliers. Accordingly, robustness can be pursued by using a robust loss as mentioned earlier. Associated with a nuclear norm penalization term, they are essentially regularized M-estimator. However, note that the LAD loss and the $L_{p}$ loss penalize the small residuals strongly and hence cannot lead to accurate prediction for unobserved entries from the trace regression viewpoint. Moreover, robust statistics reminds us that models based on the above three mentioned loss functions cannot be robust to impulsive errors [27,28]. These limitations encourage us to employ more robust surrogate loss functions to address this problem. In this paper, we present a nonconvex relaxation approach to deal with the matrix completion problem with entries heavily contaminated by noise and/or outliers.

**Figure 1:**
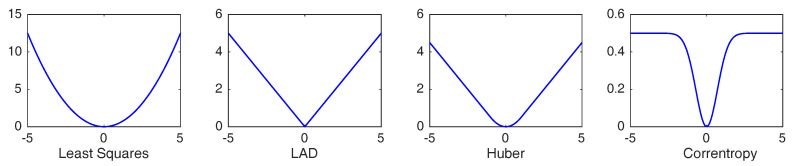
Different losses: least squares, absolute deviation loss (LAD), Huber’s loss and $\rho_{\sigma }$ (Welsch loss).

In our study, we propose the robust matrix completion model based on a robust and nonconvex loss, which is defined by:

$$\rho_{\sigma }\left(t\right)=\sigma^{2}\left(1-\exp\left(-t^{2}/\sigma^{2}\right)\right),$$

with σ>0 a scale parameter. To give an intuitive impression, plots of loss functions mentioned above are given in Figure 1. As mentioned above, this loss function is induced by the correntropy, which measures the similarity between two random variables [20,21] and has found many successful applications [29,30,31]. Recently, it was shown in [22] that regression with the correntropy-induced losses regresses towards the conditional mean function with a diverging scale parameter σ when the sample size goes to infinity. It was also shown in [32] that when the noise variable admits a unique global mode, regression with the correntropy-induced losses regresses towards the conditional mode. As argued in [22,32], learning with correntropy-induced losses can be resistant to non-Gaussian noise and outliers, while ensuring good prediction accuracy simultaneously with properly chosen σ.

Associated with the $\rho_{\sigma }$ loss, our rank-constraint robust matrix completion problem is formulated as:

(5) $$\min_{X\in R^{m\times n}}\;\;\;\;\ell_{\sigma }\left(X\right)\;s.t.\;\mathrm{rank}\left(X\right)\leq R,$$

where the data-fitting risk $\ell_{\sigma }\left(X\right)$ is given by:

$$\begin{array}{c} \ell_{\sigma }\left(X\right)=\frac{1}{2}\sum_{(i,j)\in \Omega }\rho_{\sigma }\left(X_{ij}-B_{ij}\right)=\frac{\sigma^{2}}{2}\sum_{(i,j)\in \Omega }\left(1-\exp\left(-{\left(X_{ij}-B_{ij}\right)}^{2}/\sigma^{2}\right)\right). \end{array}$$

The nuclear norm heuristic model takes the following form:

(6) $$\min_{X\in R^{m\times n}}\;\ell_{\sigma }\left(X\right)+\lambda {∥X∥}_{*},$$

where λ>0 is a regularization parameter.

### 3.2. Affine Rank Minimization Problem

In this part, we will show that our robust matrix completion approach can be extended to deal with the robust affine rank minimization problems.

It is known that the matrix completion problem (1) is a special case of the following affine rank minimization problem:

(7) $$\begin{array}{c} \min_{X\in R^{m\times n}}\;\;\mathrm{rank}\left(X\right)\;\;\;\;s.t.\;\;\;\;A\left(X\right)=b, \end{array}$$

where $b\in R^{p}$ is given, and $A:R^{m\times n}\to R^{p}$ is a linear operator defined by:

$$\begin{array}{c} A\left(\cdot \right):={\left[\langle A^{1},\cdot \rangle ,\;\langle A^{2},\cdot \rangle ,\ldots ,\;\langle A^{p},\cdot \rangle \right]}^{T}, \end{array}$$

where $A^{i}\in R^{m\times n}$ for each *i*. Introduced and studied in [33], this problem has drawn much attention in recent years [14,15,16,23]. Note that (7) can be reduced to the matrix completion problem (1) if we set p=|Ω| (the cardinality of Ω), and let $A^{(i-1)n+j}=e_{i}\left(m\right)e_{j}{\left(n\right)}^{T}$ for each (i,j)∈Ω, where $e_{i}\left(m\right),i\;=\;1,\ldots ,m$ and $e_{j}\left(n\right),j=1,\ldots ,n$ are the canonical basis vector of $R^{m}$ and $R^{n}$, respectively.

In fact, (5) and (6) can be naturally extended to handle cases with noise and outliers of (7). Denote the risk as follows:

$$\begin{array}{c} {\tilde{\ell }}_{\sigma }\left(X\right)=\frac{\sigma^{2}}{2}\sum_{i=1}^{p}\left(1-\exp\left(-{\left(\langle A^{i},X\rangle -b_{i}\right)}^{2}/\sigma^{2}\right)\right). \end{array}$$

The rank constrained model can be formulated as:

(8) $$\min_{X\in R^{m\times n}}\;{\tilde{\ell }}_{\sigma }\left(X\right)\;\;s.t.\;\;\mathrm{rank}\left(X\right)\leq R,$$

and the nuclear norm regularized heuristic takes the form:

(9) $$\begin{array}{cc} \;\;\;\; & \min_{X\in R^{m\times n}}\;\;{\tilde{\ell }}_{\sigma }\left(X\right)+\lambda {∥X∥}_{*}. \end{array}$$

Referring to computational considerations presented below, we will focus on the more general optimization problems (8) and (9), which can be directly applied to (5) and (6).

## 4. Algorithms and Analysis

We consider using gradient descent-based algorithms to solve the proposed models. It is usually admitted that gradient descent is not very efficient. However, in our experiments, we find that gradient descent is still efficient, and comparable with some state-of-the-art methods. On the other hand, we present recoverability and convergence rate results for gradient descent applied to the proposed models. Such results and analysis may help us better understand the models and such a nonconvex loss function from the algorithmic aspects.

We first consider gradient descent with hard thresholding for solving (8). The derivation is standard. Denote $S_{R}:=\left\{X\in R^{m\times n}\;|\;\mathrm{rank}\left(X\right)\leq R\right\}$. By the differentiability of $\ell_{\sigma }$, when *Y* is sufficiently close to *X*, $\ell_{\sigma }$ can be approximated by:

$$\ell_{\sigma }\left(X\right)\approx \ell_{\sigma }\left(Y\right)+\langle \nabla \ell_{\sigma }\left(Y\right),X-Y\rangle +\frac{\alpha }{2}{∥X-Y∥}_{F}^{2}.$$

Here, α>0 is a parameter, and $\nabla \ell_{\sigma }\left(Y\right)$, the gradient of $\ell_{\sigma }$ at *Y*, is equal to:

(10) $$\sum_{i=1}^{p}\exp\left(-{\left(\langle A^{i},Y\rangle -b_{i}\right)}^{2}/\sigma^{2}\right)\left(\langle A^{i},Y\rangle -b_{i}\right)A^{i}.$$

Now, the iterates can be generated as follows:

(11) $$\begin{array}{c} \;X^{\left(k+1\right)}=\arg\min_{X\in S_{R}}\ell_{\sigma }\left(X^{\left(k\right)}\right)+\langle \nabla \ell_{\sigma }\left(X^{\left(k\right)}\right),X-X^{\left(k\right)}\rangle \\ \;\;\;\;\;\;\;\;\;\;\;\;\;\;\;+\frac{\alpha }{2}{∥X-X^{\left(k\right)}∥}_{F}^{2}\\ =\arg\min_{X\in S_{R}}{∥X-Y^{\left(k+1\right)}∥}_{F}^{2} \end{array}$$

with:

(12) $$\begin{array}{c} Y^{\left(k+1\right)}=X^{\left(k\right)}-\alpha^{-1}\nabla \ell_{\sigma }\left(X^{\left(k\right)}\right). \end{array}$$

We simply write (11) as $X^{\left(k+1\right)}=P_{S_{R}}\left(Y^{\left(k+1\right)}\right)$, where $P_{S_{R}}$ denotes the hard thresholding operator, i.e., the best rank-*R* approximation to $Y^{\left(k+1\right)}$. The algorithm is presented in Algorithm 1.

**Algorithm 1** Gradient descent iterative hard thresholding for (8).
**Input:** linear operator $A:R^{m\times n}\to R^{p}$, initial guess $X^{\left(0\right)}\in R^{m\times n}$, prescribed rank R≥1, σ>0  **Output:** the recovered matrix $X^{\left(k+1\right)}$   **while** a certain stopping criterion is not satisfied **do** 1: Choose a fixed step-size $\alpha^{-1}>0$.  2: Compute the gradient descent step (12) $$Y^{\left(k+1\right)}=X^{\left(k\right)}-\alpha^{-1}\nabla \ell_{\sigma }\left(X^{\left(k\right)}\right).$$   3: Perform the hard thresholding operator to obtain $$\;X^{\left(k+1\right)}=P_{S_{R}}\left(Y^{\left(k+1\right)}\right),$$ and set k:=k+1. **end while**


The algorithm starts from an initial guess $X^{\left(0\right)}$ and continues until some stopping criterion is satisfied, e.g., $∥X^{\left(k+1\right)}-X^{\left(k\right)}{∥}_{F}\leq \epsilon$, where ϵ is a certain given positive number. Indeed, such a stopping criterion makes sense, as Proposition A3 shows that $∥X^{\left(k\right)}-X^{\left(k+1\right)}{∥}_{F}\to 0$. To ensure the convergence, the step-size should satisfy $\alpha >L:={∥A∥}_{2}^{2}$, where ${∥A∥}_{2}$ denotes the spectral norm of A. For matrix completion, the spectral norm is smaller than one, and thus, we can set α>1. In Appendix A, we have shown the Lipschitz continuity of $\nabla \ell_{\sigma }\left(\cdot \right)$, which is necessary for the convergence of the algorithm. α can also be self-adaptive by using a certain line-search rule. Algorithm 2 is the line-search version of Algorithm 1.

**Algorithm 2** Line-search version of Algorithm 1.**Input:** linear operator $A:R^{m\times n}\to R^{p}$, initial guess $X^{\left(0\right)}\in R^{m\times n}$, prescribed rank R≥1, σ>0, $\alpha^{\left(0\right)}>0$, δ∈(0,1), η>1
**Output:** the recovered matrix $X^{\left(k+1\right)}$
**while** a certain stopping criterion is not satisfied **do** 1: $\alpha^{\left(k+1\right)}=\alpha^{\left(k\right)}$
 **repeat**  2: $X^{\left(k+1\right)}=P_{S_{R}}\left(X^{\left(k\right)}-\frac{1}{\alpha^{\left(k+1\right)}}\nabla \ell_{\sigma }\left(X^{\left(k\right)}\right)\right)$  3: $\alpha^{\left(k+1\right)}:=\alpha^{\left(k+1\right)}\eta$
 **until** $\ell_{\sigma }\left(X^{\left(k+1\right)}\right)\leq \ell_{\sigma }\left(X^{\left(k\right)}\right)-\frac{\delta \alpha^{\left(k+1\right)}}{2}{∥X^{\left(k+1\right)}-X^{\left(k\right)}∥}_{F}^{2}$ 4: $\alpha^{\left(k+1\right)}:=\alpha^{\left(k+1\right)}/\eta$, and set k:=k+1. **end while**


Solving (9) is similar, with only the hard thresholding $P_{R}$ replaced by the soft thresholding $S_{\tau }$, which can be derived as follows. Denote $Y^{\left(k+1\right)}=U\mathrm{diag}\left({\left\{\sigma_{i}\right\}}_{1\leq i\leq r}\right)V^{T}$ as the SVD of $Y^{\left(k+1\right)}$. Then, $S_{\lambda /\alpha }$ is the matrix soft thresholding operator [13,16] defined as $S_{\lambda /\alpha }\left(Y^{\left(k+1\right)}\right)=U\mathrm{diag}\left(\max\{\sigma_{i}-\lambda /\alpha ,0\}\right)V^{T}.$ Gradient descent-based soft thresholding is summarized in Algorithm 3.

**Algorithm 3** Gradient descent iterative soft thresholding for (9).**Input:** linear operator $A:R^{m\times n}\to R^{p}$, initial guess $X^{\left(0\right)}\in R^{m\times n}$, parameter λ>0, σ>0**Output:** the recovered matrix $X^{\left(k+1\right)}$**while** a certain stopping criterion is not satisfied **do** 1: Choose a fixed step-size $\alpha^{-1}>0$, or choose it via the line-search rule.  2: Compute $$Y^{\left(k+1\right)}=X^{\left(k\right)}-\alpha^{-1}\nabla \ell_{\sigma }\left(X^{\left(k\right)}\right).$$ 3: Perform the soft thresholding operator to obtain $$X^{\left(k+1\right)}=S_{\lambda /\alpha }\left(Y^{\left(k+1\right)}\right),$$ and set k:=k+1. **end while**


### 4.1. Convergence

With the Lipschitz continuity of $\nabla \ell_{\sigma }$ presented in Appendix A, it is a standard routine to show the convergence of Algorithms 1 and 3, i.e., let $\left\{X^{\left(k\right)}\right\}$ be a sequence generated by Algorithm 1 or 3. Then, every limit point of the sequence is a critical point of the problem. In fact, the results can be enhanced to the statement that “the entire sequence converges to a critical point”, namely one can prove that $\lim_{k\to \infty }X^{\left(k\right)}=X^{*}$ where $X^{*}$ is a critical point. This can be achieved by verifying the so-called Kurdyka–ojasiewicz (KL) property [34] of the problems (8) and (9). As this is not the main concern of this paper, we omit the verification here.

### 4.2. Recoverability and Linear Convergence Rate

For affine rank minimization problems, the convergence rate results have been obtained in the literature; see, e.g., [23,24]. However, all the existing results are obtained for algorithms that solve the optimization problems incorporating the least squares loss. In this part, we are concerned with the recoverability and convergence rate of Algorithm 1. These results give the understanding of this loss function from the algorithmic aspect, which is in accordance with and extends our previous work [22].

It has been known that the convergence rate analysis requires the matrix RIPcondition [33]. In our context, instead of using the matrix RIP, we adopt the concept of the matrix scalable restricted isometry property (SRIP) [24].

**Definition** **1** (SRIP [24]). *For any $X\in S_{r}$, there exist constants $\nu_{r},\mu_{r}>0$ such that:*

$$\nu_{r}{∥X∥}_{F}\leq {∥A\left(X\right)∥}_{F}\leq \mu_{r}{∥X∥}_{F}.$$

Due to the scalability of $\nu_{r},\mu_{r}$ on the operator A, SRIP is a generalization of the RIP [33] as commented in [24]. We point out that the results of Algorithm 1 for the affine rank minimization problem (8) rely on the SRIP condition. However, in the matrix completion problem (5), this condition cannot be met, since $\nu_{r}$ in this case is zero. Consequently, the results provided below cannot be applied directly to the matrix completion problem (5). However, similar results might be established for (5), if some refined RIP conditions are assumed to hold for the operator A in the situation of matrix completion [23]. To obtain the convergence rate results, besides the SRIP condition, we also need to make some assumptions.

**Assumption** **1.**

1. *At the (k+1)-th iteration of Algorithm 1, the parameter $\sigma^{k+1}$ in the loss function $\ell_{\sigma }$ is chosen as:*
  $$\begin{array}{c} \sigma^{k+1}=\max\left\{\frac{∥A\left(X^{\left(k\right)}\right){-b∥}_{F}}{\sqrt{2(1-\beta )}},\hat{\sigma }\right\}, \end{array}$$
  *where β∈[0.988,1), and $\hat{\sigma }$ is a positive constant.*
2. The spectral norm of A is upper bounded as ${∥A∥}_{2}^{2}\leq \frac{6}{5}\nu_{2R}^{2}.$

Based on Assumption 1, the following results for Algorithm 1 can be derived.

**Theorem** **1.** *Assume that $A(X^{*})+\epsilon =b$, where $X^{*}$ is the matrix to be recovered and $\mathrm{rank}(X^{*})=R$. Assume that Assumption 1 holds. Let $\left\{X^{\left(k\right)}\right\}$ be generated by Algorithm 1, with the step-size $\alpha ={∥A∥}_{2}^{2}$. Then*

1. *at iteration (k+1), Algorithm 1 will recover a matrix $X^{k+1}$ satisfying:*
  $$∥X^{\left(k+1\right)}-X^{*}{∥}_{F}\leq q_{1}^{k+1}{∥X^{\left(0\right)}-X^{*}∥}_{F}+\frac{2}{1-q_{1}}\frac{{∥\epsilon ∥}_{F}}{{∥A∥}_{2}},$$
  *where $q_{1}\in \left(0.8165,0.9082\right)$ depending on β.*
2. *If there is no noise or outliers, i.e., $A(X^{*})=b$, then the algorithm converges linearly in the least squares and $\ell_{\sigma }$ sense, respectively, i.e.,*
  $$\begin{array}{ccc} ∥A\left(X^{\left(k+1\right)}\right){-b∥}^{2} & \leq & q_{2}{∥A\left(X^{\left(k\right)}\right)-b∥}^{2},\;\mathrm{and}\\ {\tilde{\ell }}_{\sigma^{k+1}}\left(X^{\left(k+1\right)}\right) & \leq & q_{3}\;{\tilde{\ell }}_{\sigma^{k}}\left(X^{\left(k\right)}\right), \end{array}$$
  *where $q_{2}\in \left(0.8,0.9898\right)$ and $q_{3}\in \left(0.2,0.262\right)$, depending on the choice of β.*

The proof of Theorem 1 relies on the following lemmas, which reveal certain properties of the loss function ${\tilde{\ell }}_{\sigma }$.

**Lemma** **1.** *For any σ>0 and t∈R, it holds:*

$$\begin{array}{c} \frac{\sigma^{2}}{2}\left(1-\exp\left(\frac{-t^{2}}{\sigma^{2}}\right)\right)\leq \frac{t^{2}}{2}. \end{array}$$

**Proof.**  For any σ>0, let $f\left(t\right):=\frac{t^{2}}{2}-\frac{\sigma^{2}}{2}\left(1-\exp\left(\frac{-t^{2}}{\sigma^{2}}\right)\right)$. Since f(t) is even, we need to only consider t≥0. Note that $f^{{}^{\prime }}\left(t\right)=t-t\exp\left(\frac{-t^{2}}{\sigma^{2}}\right)$, which is nonnegative when t≥0. Therefore, f(t) is a nondecreasing function on [0,+∞). On the other hand, $f^{{}^{\prime }}\left(0\right)=0$ and f(t)=0. Thus, the minimum of f(t) is f(0)=0. As a result, f(t)≥0. This completes the proof. ☐

**Lemma** **2.** *Assuming that β∈[0,1), and 0<δ≤2(1−β), it holds:*

$$\begin{array}{c} g(\delta ):=1-\exp(-\delta )-\beta \delta \geq 0. \end{array}$$

**Proof.**  Since δ>0, it is not hard to check that $1-\exp\left(-\delta \right)\geq \delta -\frac{1}{2}\delta^{2}$. From the range of δ, it follows $\delta -\frac{1}{2}\delta^{2}\geq \beta \delta$. This completes the proof. ☐

**Lemma** **3.** Given a fixed t∈R, for σ>0, $h\left(\sigma \right):=\sigma^{2}\left(1-\exp\left(-t^{2}/\sigma^{2}\right)\right)$ is nondecreasing with respect to σ.

**Proof.**  It is not hard to check that $h^{\prime }$ is nonnegative on σ>0. ☐

**Proof** **of** **Theorem** **1.** By the fact that $X^{*}$ is rank-*R* and $X^{\left(k+1\right)}$ is the best rank-*R* approximation to $Y^{\left(k+1\right)}$, we have:

$$\begin{array}{cc} & ∥X^{\left(k+1\right)}-X^{*}{∥}_{F}\\ \leq & ∥X^{\left(k+1\right)}-Y^{\left(k+1\right)}{∥}_{F}+{∥Y^{\left(k+1\right)}-X^{*}∥}_{F}\\ \leq & 2∥Y^{\left(k+1\right)}-X^{*}{∥}_{F}\\ = & 2∥X^{\left(k\right)}-X^{*}-\frac{1}{\alpha }\nabla \ell_{\sigma^{k+1}}\left(X^{\left(k\right)}\right){∥}_{F}. \end{array}$$

Since:

$$\begin{array}{ccc} \mathrm{vec}\left(\nabla \ell_{\sigma^{k+1}}\left(X^{\left(k\right)}\right)\right) & = & A^{T}\Lambda \left(A\mathrm{vec}(X^{\left(k\right)})-b\right)\\ & = & A^{T}\Lambda \left(A\mathrm{vec}(X^{\left(k\right)}-X^{*})-\epsilon \right), \end{array}$$

we know that:

$$\begin{array}{cc} & {∥X^{\left(k\right)}-X^{*}-\frac{1}{\alpha }\nabla \ell_{\sigma^{k+1}}\left(X^{\left(k\right)}\right)∥}_{F}\\ = & {∥\mathrm{vec}\left(X^{\left(k\right)}-X^{*}\right)-\frac{1}{\alpha }A^{T}\Lambda \left(A\mathrm{vec}(X^{\left(k\right)}-X^{*})-\epsilon \right)∥}_{F}\\ \leq & {∥\mathrm{vec}\left(X^{\left(k\right)}-X^{*}\right)-\frac{1}{\alpha }A^{T}\Lambda A\mathrm{vec}\left(X^{\left(k\right)}-X^{*}\right)∥}_{F}\\ & +\frac{1}{\alpha }{∥A^{T}\Lambda \epsilon ∥}_{F}\\ \leq & {∥\mathrm{vec}\left(X^{\left(k\right)}-X^{*}\right)-\frac{1}{\alpha }A^{T}\Lambda A\mathrm{vec}\left(X^{\left(k\right)}-X^{*}\right)∥}_{F}\\ & +\frac{{∥\epsilon ∥}_{F}}{{∥A∥}_{2}}, \end{array}$$

where the last inequality follows from:

$$∥A^{T}{\Lambda \epsilon ∥}_{F}\leq {∥A∥}_{2}{∥\Lambda ∥}_{2}{∥\epsilon ∥}_{F}\leq {∥A∥}_{2}{∥\epsilon ∥}_{F}$$

and the choice of the step-size α. It remains to estimate $∥\mathrm{vec}\left(X^{\left(k\right)}-X^{*}\right)-\frac{1}{\alpha }A^{T}\Lambda A\mathrm{vec}\left(X^{\left(k\right)}-X^{*}\right){∥}_{F}$. We first see that:

(13) $$\begin{array}{cc} & {∥\mathrm{vec}\left(X^{\left(k\right)}-X^{*}\right)-\frac{1}{\alpha }A^{T}\Lambda A\mathrm{vec}\left(X^{\left(k\right)}-X^{*}\right)∥}_{F}^{2}\\ = & -\frac{2}{\alpha }\langle \mathrm{vec}\left(X^{\left(k\right)}-X^{*}\right),A^{T}\Lambda A\mathrm{vec}\left(X^{\left(k\right)}-X^{*}\right)\rangle \\ & +\frac{1}{\alpha^{2}}{∥A^{T}\Lambda A\mathrm{vec}\left(X^{\left(k\right)}-X^{*}\right)∥}_{F}^{2}+{∥X^{\left(k\right)}-X^{*}∥}_{F}^{2} \end{array}$$

To verify our first assertion, it remains to bound the first two terms by means of $∥X^{\left(k\right)}-X^{*}{∥}_{F}^{2}$. We consider the first term. Denoting $y_{i}^{k}=\langle A^{i},X^{\left(k\right)}-X^{*}\rangle$, we know that:

$$\begin{array}{cc} & \langle \mathrm{vec}\left(X^{\left(k\right)}-X^{*}\right),A^{T}\Lambda A\mathrm{vec}\left(X^{\left(k\right)}-X^{*}\right)\rangle \\ = & \langle A\mathrm{vec}\left(X^{\left(k\right)}-X^{*}\right),\Lambda A\mathrm{vec}\left(X^{\left(k\right)}-X^{*}\right)\rangle \\ = & \sum_{i=1}^{p}\exp\left(-{\left(\frac{\langle A^{i},X^{\left(k\right)}\rangle -b_{i}}{\sigma^{k+1}}\right)}^{2}\right){\left(y_{i}^{k}\right)}^{2}. \end{array}$$

The choice of $\sigma^{k+1}$ tells us that:

$$\exp\left(-{\left(\frac{\langle A^{i},X^{\left(k\right)}\rangle -b_{i}}{\sigma^{k+1}}\right)}^{2}\right)\geq \exp\left(-2(1-\beta )\right),$$

and consequently:

(14) $$\begin{array}{c} -\frac{2}{\alpha }\langle \mathrm{vec}\left(X^{\left(k\right)}-X^{*}\right),A^{T}\Lambda A\mathrm{vec}\left(X^{\left(k\right)}-X^{*}\right)\rangle \\ \leq -\frac{2}{\alpha }\exp\left(-2(1-\beta )\right){∥A\mathrm{vec}\left(X^{\left(k\right)}-X^{*}\right)∥}_{F}^{2}. \end{array}$$

Then, by the fact that ${∥\Lambda ∥}_{2}^{2}\leq 1$ and the choice of the step-size α, we observe that the second term of (13) can be upper bounded by:

(15) $$\frac{1}{\alpha^{2}}∥A^{T}\Lambda A\mathrm{vec}\left(X^{\left(k\right)}-X^{*}\right){∥}_{F}^{2}\leq \frac{1}{\alpha }{∥A\mathrm{vec}\left(X^{\left(k\right)}-X^{*}\right)∥}_{F}^{2}.$$

Combining (14) and (15) and denoting $\gamma =1-2\exp\left(-2(1-\beta )\right)$, we come to the following conclusion:

$$\begin{array}{cc} & {∥\mathrm{vec}\left(X^{\left(k\right)}-X^{*}\right)-\frac{1}{\alpha }A^{T}\Lambda A\mathrm{vec}\left(X^{\left(k\right)}-X^{*}\right)∥}_{F}^{2}\\ \leq & {∥X^{\left(k\right)}-X^{*}∥}_{F}^{2}+\frac{\gamma }{\alpha }{∥A\mathrm{vec}(X^{\left(k\right)}-X^{*})∥}_{F}^{2}\\ \leq & {∥X^{\left(k\right)}-X^{*}∥}_{F}^{2}+\frac{\gamma \nu_{2R}^{2}}{\alpha }{∥X^{\left(k\right)}-X^{*}∥}_{F}^{2}, \end{array}$$

where the last inequality follows from the SRIP condition and the fact that γ<0 by the range of β. As a result, we get the following estimation:

(16) $$\begin{array}{ccc} ∥X^{\left(k+1\right)}-X^{*}{∥}_{F} & \leq & 2∥X^{\left(k\right)}-X^{*}-\frac{1}{\alpha }\nabla \ell_{\sigma^{k+1}}\left(X^{\left(k\right)}\right){∥}_{F}\\ & \leq & 2\sqrt{1+\frac{\gamma \nu_{2R}^{2}}{\alpha }}{∥X^{\left(k\right)}-X^{*}∥}_{F}+2\frac{{∥\epsilon ∥}_{F}}{{∥A∥}_{2}}\\ & \leq & 2\sqrt{1+\frac{5\gamma }{6}}{∥X^{\left(k\right)}-X^{*}∥}_{F}+2\frac{{∥\epsilon ∥}_{F}}{{∥A∥}_{2}} \end{array}$$

where the last inequality follows from the assumption $\alpha ={∥A∥}_{2}^{2}\leq 6/5\nu_{2R}^{2}$. Denote $q_{1}=2\sqrt{1+\frac{5\gamma }{6}}$. The range of β tells us that $q_{1}\in \left(0.8165,0.9082\right).$ Iterating (16), we obtain:

$$∥X^{\left(k+1\right)}-X^{*}{∥}_{F}\leq q_{1}^{k+1}{∥X^{\left(0\right)}-X^{*}∥}_{F}+\frac{2}{1-q_{1}}\frac{{∥\epsilon ∥}_{F}}{{∥A∥}_{2}}.$$

Therefore, The first assertion concerning the recoverability is proven.

Suppose there is no noise or outliers, i.e., we have $A(X^{*})=b$. In this case, it follows from (16) that:

$$\begin{array}{c} ∥X^{\left(k+1\right)}-X^{*}{∥}_{F}\leq q_{1}{∥X^{\left(k\right)}-X^{*}∥}_{F}, \end{array}$$

and then, the SRIP condition tells us that:

$$\begin{array}{ccc} ∥A\left(X^{k}\right){-b∥}_{F}^{2} & \leq & \mu_{2R}^{2}{∥X^{k+1}-X^{*}∥}_{F}^{2}\\ & \leq & \mu_{2R}^{2}q_{1}^{2}{∥X^{\left(k\right)}-X^{*}∥}_{F}^{2}\\ & \leq & {\left(\frac{\mu_{2R}}{\nu_{2R}}\right)}^{2}q_{1}^{2}{∥A\left(X^{k}\right)-b∥}_{F}^{2}\\ & \leq & \frac{6}{5}q_{1}^{2}{∥A\left(X^{k}\right)-b∥}_{F}^{2}, \end{array}$$

where the last inequality comes from the inequality chain $\mu_{2R}^{2}\leq {∥A∥}_{2}^{2}\leq 6/5\nu_{2R}^{2}$. Denote $q_{2}=6q_{1}^{2}/5$. Then, $q_{2}\in \left(0.8,0.9898\right)$. Therefore, the algorithm converges linearly to $X^{*}$ in the least squares sense.

We now proceed to show the linear convergence in the ${\tilde{\ell }}_{\sigma }$ sense. Following from the inequality $∥X^{\left(k+1\right)}-Y^{\left(k+1\right)}{∥}_{F}^{2}\leq {∥X^{*}-Y^{\left(k+1\right)}∥}_{F}^{2}$, we obtain:

$$\begin{array}{cc} & \frac{\alpha }{2}{∥X^{\left(k+1\right)}-X^{\left(k\right)}∥}_{F}^{2}\\ & +\langle \nabla \ell_{\sigma^{k+1}}\left(X^{\left(k\right)}\right),X^{\left(k+1\right)}-X^{\left(k\right)}\rangle \\ \leq & \frac{\alpha }{2}{∥X^{\left(k\right)}-X^{*}∥}_{F}^{2}+\langle \nabla \ell_{\sigma^{k+1}}\left(X^{\left(k\right)}\right),X^{*}-X^{\left(k\right)}\rangle . \end{array}$$

Combining with Inequality (A1), we see that ${\tilde{\ell }}_{\sigma^{k+1}}\left(X^{\left(k+1\right)}\right)$ can be upper bounded by:

(17) $${\tilde{\ell }}_{\sigma^{k+1}}\left(X^{\left(k\right)}\right)+\frac{\alpha }{2}{∥X^{\left(k\right)}-X^{*}∥}_{F}^{2}+\langle \nabla {\tilde{\ell }}_{\sigma^{k+1}}\left(X^{\left(k\right)}\right),X^{*}-X^{\left(k\right)}\rangle .$$

We need to upper bound $\langle \nabla {\tilde{\ell }}_{\sigma^{k+1}}\left(X^{\left(k\right)}\right),X^{*}-X^{\left(k\right)}\rangle$ and $\frac{\alpha }{2}{∥X^{\left(k\right)}-X^{*}∥}_{F}^{2}$ in terms of ${\tilde{\ell }}_{\sigma^{k+1}}\left(X^{\left(k\right)}\right)$. We first consider the second term. Under the SRIP condition, we have:

$$\begin{array}{ccc} ∥X^{\left(k\right)}-X^{*}{∥}_{F}^{2} & \leq & \frac{1}{\nu_{2R}^{2}}{∥A\left(X^{\left(k\right)}-X^{*}\right)∥}_{F}^{2}\\ & = & \frac{1}{\nu_{2R}^{2}}{∥A\left(X^{\left(k\right)}\right)-b∥}_{F}^{2}. \end{array}$$

By setting $\delta ={\left(\frac{y_{i}^{k}}{\sigma^{k+1}}\right)}^{2}$, we get δ≤2(1−β). Lemma 2 tells us that:

$$\begin{array}{c} \beta {\left(y_{i}^{k}\right)}^{2}\leq {\left(\sigma^{k+1}\right)}^{2}\left(1-\exp\left(-{\left(y_{i}^{k}/\sigma^{k+1}\right)}^{2}\right)\right). \end{array}$$

Summing the above inequalities over *i* from 1 to *p*, we have:

$$\begin{array}{cc} & \beta ∥A\left(X^{\left(k\right)}\right){-b∥}_{F}^{2}\\ \leq & {\left(\sigma^{k+1}\right)}^{2}\sum_{i=1}^{p}\left(1-\exp\left(-{\left(y_{i}^{k}/\sigma^{k+1}\right)}^{2}\right)\right)\\ = & 2{\tilde{\ell }}_{\sigma^{k+1}}\left(X^{\left(k\right)}\right). \end{array}$$

Therefore, $\frac{\alpha }{2}{∥X^{\left(k\right)}-X^{*}∥}_{F}^{2}$ can be bounded as follows:

(18) $$\begin{array}{ccc} \frac{\alpha }{2}{∥X^{\left(k\right)}-X^{*}∥}_{F}^{2} & \leq & \frac{\alpha }{2\nu_{2R}^{2}}{∥A\left(X^{\left(k\right)}-X^{*}\right)∥}^{2}\\ & \leq & \frac{\alpha }{\beta \nu_{2R}^{2}}{\tilde{\ell }}_{\sigma^{k+1}}\left(X^{\left(k\right)}\right). \end{array}$$

We proceed to bound $\langle \nabla {\tilde{\ell }}_{\sigma^{k+1}}\left(X^{\left(k\right)}\right),X^{*}-X^{\left(k\right)}\rangle$. It follows from (14) and Lemma 1 that:

(19) $$\begin{array}{c} \;\;\;\langle \nabla {\tilde{\ell }}_{\sigma^{k+1}}\left(X^{\left(k\right)}\right),X^{*}-X^{\left(k\right)}\rangle \\ \leq -\exp\left(-2\left(1-\beta \right)\right)∥A\left(X^{\left(k\right)}\right){-b∥}^{2}\\ \leq -2\exp\left(-2\left(1-\beta \right)\right){\tilde{\ell }}_{\sigma^{k+1}}\left(X^{\left(k\right)}\right). \end{array}$$

Combining (17)–(19) together, we get:

$$\begin{array}{cc} & {\tilde{\ell }}_{\sigma^{k+1}}\left(X^{\left(k+1\right)}\right)\\ \leq & \left(1+\frac{\alpha }{\beta \nu_{2R}^{2}}-2\exp\left(-2\left(1-\beta \right)\right)\right){\tilde{\ell }}_{\sigma^{k+1}}\left(X^{\left(k\right)}\right)\\ \leq & \left(1+\frac{6}{5\beta }-2\exp\left(-2\left(1-\beta \right)\right)\right){\tilde{\ell }}_{\sigma^{k+1}}\left(X^{\left(k\right)}\right), \end{array}$$

where the last inequality follows from $\alpha \leq \frac{6}{5}\nu_{2R}^{2}$.

By Lemma 3, the function $\sigma^{2}\left(1-\exp\left(-t^{2}/\sigma^{2}\right)\right)$ is nondecreasing with respect to σ>0. This in connection with the fact that:

$$\begin{array}{cc} & \sigma^{k+1}=\max\left\{\frac{∥A\left(X^{\left(k\right)}\right){-b∥}_{F}}{\sqrt{2(1-\beta )}},\sigma \right\}\\ \leq & \sigma^{k}=\max\left\{\frac{∥A\left(X^{\left(k-1\right)}\right){-b∥}_{F}}{\sqrt{2(1-\beta )}},\sigma \right\} \end{array}$$

yields ${\tilde{\ell }}_{\sigma^{k+1}}\left(X^{\left(k\right)}\right)\leq {\tilde{\ell }}_{\sigma^{k}}\left(X^{\left(k\right)}\right)$. Let $q_{3}=1+\frac{6}{5\beta }-2\exp\left(-2(1-\beta )\right)$, and consequently, $q_{3}\;\in \;\left(0.2,0.2620\right)$. We thus have:

$${\tilde{\ell }}_{\sigma^{k+1}}\left(X^{\left(k+1\right)}\right)\leq q_{3}{\tilde{\ell }}_{\sigma^{k+1}}\left(X^{\left(k\right)}\right)\leq q_{3}\;{\tilde{\ell }}_{\sigma^{k}}\left(X^{\left(k\right)}\right).$$

The proof is now completed. ☐

The above results show that it is possible that Algorithm 1 will find $X^{*}$ if the magnitude of the noise is not too large. Moreover, the results also imply that the algorithm is safe when there is no noise.

## 5. Numerical Experiments

This section presents numerical experiments to illustrate the effectiveness of our methods. Empirical comparisons with other methods are implemented on synthetic and real data contaminated by outliers or non-Gaussian noise.

The following 4 algorithms are implemented. RMC-$\ell_{\sigma }$-IHTand RMC-$\ell_{\sigma }$-ISTare denoted as Algorithms 1 and 3 incorporated with the line-search rule, respectively. The approach proposed in [16] is denoted as MC-$\ell_{2}$-IST, which is an iterative soft thresholding algorithm based on the least squares loss. The robust approach based on the LAD loss proposed in [17] is denoted by RMC-$\ell_{1}$-ADM. Empirically, the σ value of $\ell_{\sigma }$ is set to be 0.5; the tuned parameter λ of RMC-$\ell_{\sigma }$-IST and MC-$\ell_{2}$-IST is set to $\lambda =\frac{\min\{m,n\}}{10\sqrt{\max\{m,n\}}}$, while for RMC-$\ell_{1}$-ADM, $\lambda =1/\sqrt{\max\{m,n\}}$, as suggested in [17]. All the numerical computations are conducted on an Intel i7-3770 CPU desktop computer with 16 GB of RAM. The supporting software is MATLAB R2013a. Some notations used frequently in this section are introduced first in Table 1. Bold number in the tables of this section means that it is the best among the competitors.

### 5.1. Evaluation on Synthetic Data

The synthetic datasets are generated in the following way:

1. Generating a low rank matrix: We first generate an m×n matrix with i.i.d. Gaussian entries ∼*N*(0,1), where m=n=1000. Then, a $\left\lfloor \rho_{r}m\right\rfloor$-rank matrix *M* is obtained from the above matrix by rank truncation, where $\rho_{r}$ varies from 0.04–0.4.
2. Adding outliers: We create a zero matrix $E\in R^{m\times n}$ and uniformly randomly sample $\rho_{o}m^{2}$ entries, where $\rho_{o}$ varies from 0–0.6. These entries are randomly drawn from the chi-square distribution, with four degrees of freedom. Multiplied by 10, the matrix *E* is used as the sparse error matrix.
3. Missing entries: $\rho_{m}m^{2}$ of the entries are randomly missing, with $\rho_{m}$ varying between {0,10%,20%,30%}. Finally, the observed matrix is denoted as $B=P_{\Omega }\left(M+E\right)$.

RMC-$\ell_{\sigma }$-IHT (Algorithm 1), RMC-$\ell_{\sigma }$-IST (Algorithm 3) and RMC-$\ell_{1}$-ADM [17] are implemented respectively on the matrix completion problem with the datasets generated above. For these three algorithms, the same initial guess with the all-zero matrix $X^{0}=0$ is applied. The stopping criterion is $∥X^{\left(k+1\right)}-X^{\left(k\right)}{∥}_{F}\leq 10^{-3}$, or restrictions on the number of iterations, which is set to be 500. For each tuple $\left(\rho_{m},\rho_{r},\rho_{o}\right)$, we repeat 10 runs. The algorithm is regarded as successful if the relative error of the result $\hat{X}$ satisfies $∥\hat{X}{-M∥}_{F}/{∥M∥}_{F}\leq 10^{-1}$.

**Figure 2:**
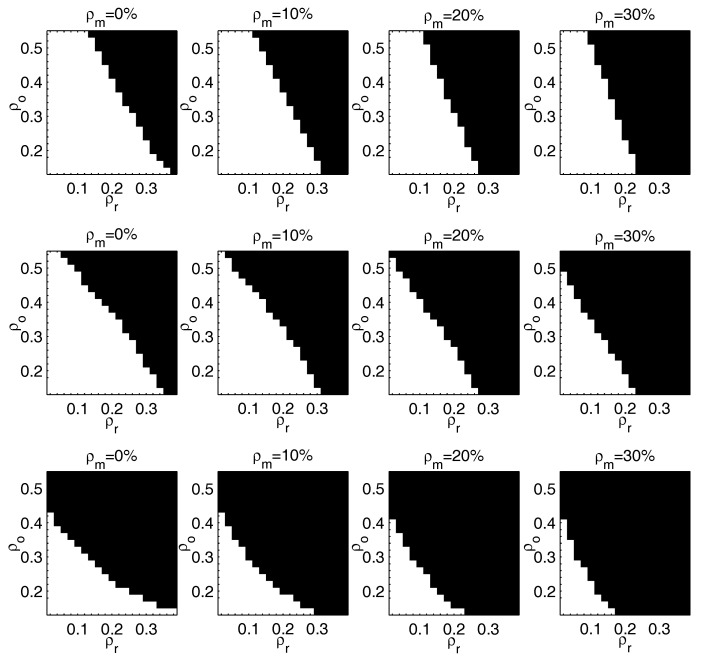
Phase transition diagrams of RMC-$\ell_{\sigma }$-IHT (Algorithm 1), RMC-$\ell_{\sigma }$-IST (Algorithm 3) and RMC-$\ell_{1}$-ADM [17]. The first row: RMC-$\ell_{\sigma }$-IHT; the second row: RMC-$\ell_{\sigma }$-IST; the last row: RMC-$\ell_{1}$-ADM. *x*-axis: $\rho_{r}\in \left[0.04,0.4\right]$; *y*-axis: $\rho_{o}\in \left[0,0.6\right]$. From the first column to the last column, $\rho_{m}$ varies from 0–30%.

Experimental results of RMC-$\ell_{\sigma }$-IHT (top), RMC-$\ell_{\sigma }$-IST (middle) and RMC-$\ell_{1}$-ADM (bottom) are reported in Figure 2, which are given in terms of phase transition diagrams. In Figure 2, the white zones denote perfect recovery in all the experiments, while the black ones denote failure for all the experiments. In each diagram, the *x*-axis represents the ratio of rank, i.e., we let $\rho_{r}=\frac{\mathrm{rank}}{m}\in \left[0.04,0.4\right]$, and the *y*-axis represents the level of outliers, i.e., we let $\rho_{o}=\frac{♯\mathrm{outliers}}{m^{2}}\in \left[0,0.6\right]$. The level of missing entries $\rho_{m}$ varies from left to right in each row. As shown in Figure 2, our approach outperforms RMC-$\ell_{1}$-ADM when $\rho_{o}$ and $\rho_{r}$ increase. We also observe that RMC-$\ell_{\sigma }$-IHT performs better than RMC-$\ell_{\sigma }$-IST when the level of outliers increases, while RMC-$\ell_{\sigma }$-IST outperforms RMC-$\ell_{\sigma }$-IHT when the ratio of missing entries increases.

Comparison of the computational time and the relative error are also reported in Table 2. In this experiment, the level of missing entries $\rho_{m}=\left\{20\%,30\%\right\}$, the ratio of rank $\rho_{r}=0.1$ and the level of outliers $\rho_{o}$ varies between {0.1,0.15,0.2,0.25,0.3}. For each $\rho_{o}$, we randomly generate 20 instances and then average the results. In the table, “time” denotes the CPU time, with the unit being second, and “rel.err” represents the relative error introduced in the previous paragraph. The results also demonstrate the improved performance of our methods in most of the cases on CPU time and relative error, especially for RMC-$\ell_{\sigma }$-IHT.

### 5.2. Image Inpainting and Denoising

One typical application of matrix completion is the image inpainting problem [4]. The datasets and the experiment are conducted as follows:

1. We first choose five gray images, named “Baboon”, “Camera Man”, “Lake”, “Lena” and “Pepper” (the size of each image is 512×512), each of which is stored in a matrix *M*.
2. The outliers matrix *E* is added to each *M*, where *E* is generated in the same way as the previous experiment, and the level of outliers $\rho_{o}$ varies among {0.3,0.4,0.5,0.6,0.7}.
3. The ratio of the missing entries is set to 30%. RMC-$\ell_{\sigma }$-IST, RMC-$\ell_{1}$-ADM and MC-$\ell_{2}$-IST, are tested in this experiment. In addition, we also test the Cauchy loss-based model $\min_{X}\ell_{c}\left(X\right)+\lambda {∥X∥}_{*}$, which is denoted as RMC-$\ell_{c}$-IST, where:
  $$\ell_{c}:=\frac{c^{2}}{2}\sum_{(i,j)\in \Omega }\ln\left(1+{\left(X_{ij}-B_{ij}\right)}^{2}/c^{2}\right),$$
  where c>0 is a parameter controlling the robustness. Empirically, we set c=0.15. Other parameters are set to the same as those of RMC-$\ell_{\sigma }$-IST. The above model is also solved by soft thresholding similar to Algorithm 3. Note that Cauchy loss has a similar shape as that of Welsch loss and also enjoys the redescending property; such a loss function is also frequently used in the robust statistics literature. The initial guess is $X^{0}=0$. The stopping criterion is $∥X^{\left(k+1\right)}-X^{\left(k\right)}{∥}_{F}\leq 10^{-2}$, or the iterations exceed 500.

Detailed comparison results in terms of the relative error and CPU time are listed in Table 3, from which one can see the efficiency of our method. Indeed, experimental results show that our method can be terminated within 80 iterations. According to the relative error in Table 3, our method performs the best in almost all cases, followed by RMC-$\ell_{c}$-IST. This is not surprising because the Cauchy loss-based model enjoys similar properties as the proposed model. We also observe that the RMC-$\ell_{1}$-ADM algorithm cannot deal with situations when images are heavily contaminated by outliers. This illustrates the robustness of our method.

**Figure 3:**
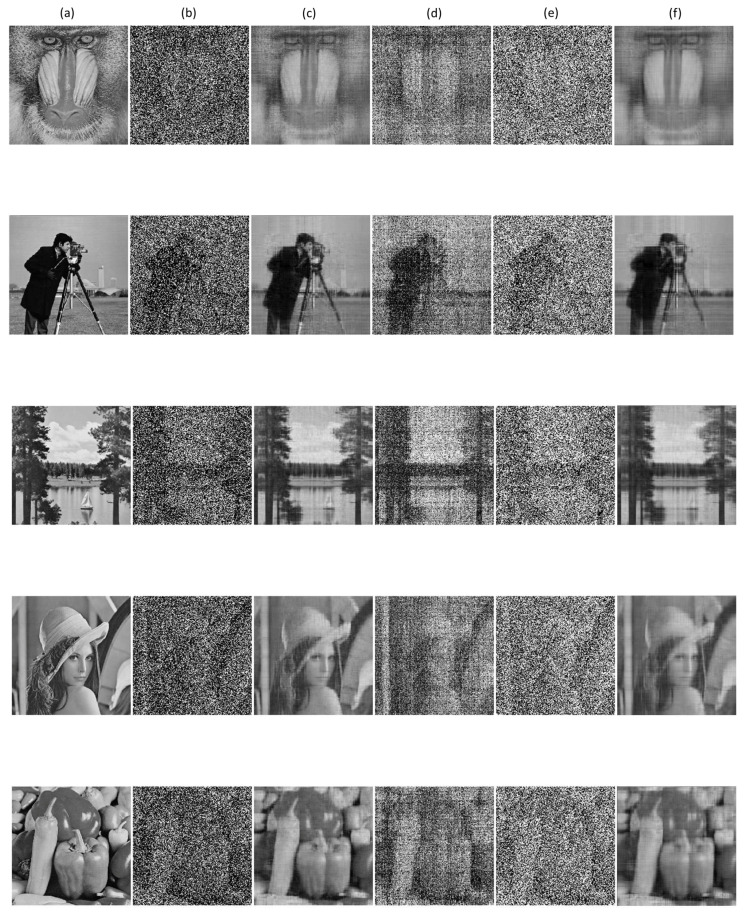
Comparison of RMC-$\ell_{\sigma }$-IST, RMC-$\ell_{1}$-ADM and MC-$\ell_{2}$-IST on different images with 60% outliers and 30% missing entries. (**a**) The original low rank images; (**b**) images with 30% missing entries and contaminated by 70% outliers; (**c**) images recovered by RMC-$\ell_{\sigma }$-IST (Algorithm 3); (**d**) images recovered by RMC-$\ell_{1}$-ADM [17]; (**e**) images recovered by MC-$\ell_{2}$-IST [16]; (**f**) images recovered by RMC-$\ell_{c}$-IST.

To better illustrate the robustness of our method empirically, we also attach images recovered by the three methods in Figure 3. For the sake of saving space, we merely list the recovery results for the case $\rho_{o}=0.6$ with 30% missing entries. In Figure 3, the first column represents five original images, namely, “Baboon”, “Camera Man”, “Lake”, “Lena” and “Pepper”. Images in the second column are contaminated images with 60% outliers and 30% missing entries. Recovered results of each image are report in the remaining columns respectively by using RMC-$\ell_{\sigma }$-IST, RMC-$\ell_{1}$-ADM, MC-$\ell_{2}$-IST and RMC-$\ell_{c}$-IST. One can observe that the images recovered by our method retain most of the important information, followed by RMC-$\ell_{c}$-IST.

**Figure 4:**
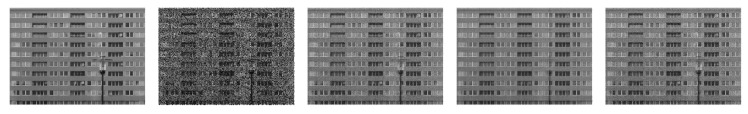
Recovery results of RMC-$\ell_{\sigma }$-IST (third), RMC-$\ell_{1}$-ADM (fourth) and MC-$\ell_{2}$-IST (fifth) on the image “Building” contaminated by non-Gaussian noise with $s_{n}=0.05$ and 30% missing entries.

Our next experiment is designed to show the effectiveness of our method in dealing with the non-Gaussian noise. We assume that the entries of the noise matrix *E* are i.i.d drawn from Student’s *t* distribution, with three degrees of freedom. We then scale *E* by a factor $s_{n}$, and we denote the corresponding $E:=s_{n}\cdot E$. The noise scale factor $s_{n}$ varies in {0.01,0.05,0.1}, and $\rho_{m}$ varies in {0.1,0.3,0.5}. The results are shown in Table 4, where the image “Building” is used. We list the recovered images in Figure 4 with the case $s_{n}=0.05$. From the table and the recovered images, we can see that our method also performs well when the image is only contaminated by non-Gaussian noise.

### 5.3. Background Subtraction

Background subtraction, also known as foreground detection, is one of the major tasks in computer vision, which aims at detecting changes in image or video sequences and finds application in video surveillance, human motion analysis and human-machine interaction from static cameras [35].

Given a sequence of images, one can cast them into a matrix *B* by vectorizing each image and then stacking row by row. In many cases, it is reasonable to assume that the background varies little. Consequently, the background forms a low rank matrix *M*, while the foreground activity is spatially localized and can be seen as the error matrix *E*. Correspondingly, the image sequence matrix *B* can be expressed as the sum of a low rank background matrix *M* and a sparse error matrix *E*, which represents the activity in the scene.

In practice, it is reasonable to assume that some entries of the image sequence are missing and the images are contaminated by noise or outliers. Therefore, the foreground object detection problem can be formulated as a robust matrix completion problem. Ref. [36] proposed to use the LAD-loss-based matrix completion approach to separate *M* and *E*. The data of this experiment were downloaded from http://perception.i2r.a-star.edu.sg/bkmodel/bkindex.html.

Our experiment in this scenario is implemented as follows:

1. We choose the sequence named “Restaurant” for our experiment, which consists of 3057 color images. Each image of “Restaurant” is 160×120 in size. From the sequence, we pick 100 continuous images and convert them to gray images to form the original matrix *B*, which is 100×19200 in size, where each row is a vector converted from an image.
2. Two types of non-Gaussian noise are added to *B*. The first type of noise is drawn from the chi-square distribution, with four degree of freedom; the second type of noise is drawn from Student’s *t* distribution, with three degrees of freedom. Then, the two types of noise are simultaneously rescaled by $s_{n}=\left\{0.01,0.02,0.05\right\}$. The last 50% of the entries are missing randomly.
3. RMC-$\ell_{\sigma }$-IHT and RMC-$\ell_{1}$-ADM are used to deal with this problem. We set R=1 in RMC-$\ell_{\sigma }$-IHT. The initial guess is the zero matrix. The stopping criterion is $∥X^{\left(k+1\right)}-X^{\left(k\right)}{∥}_{F}\leq 10^{-2}$, or the iterations exceed 200.

**Figure 5:**
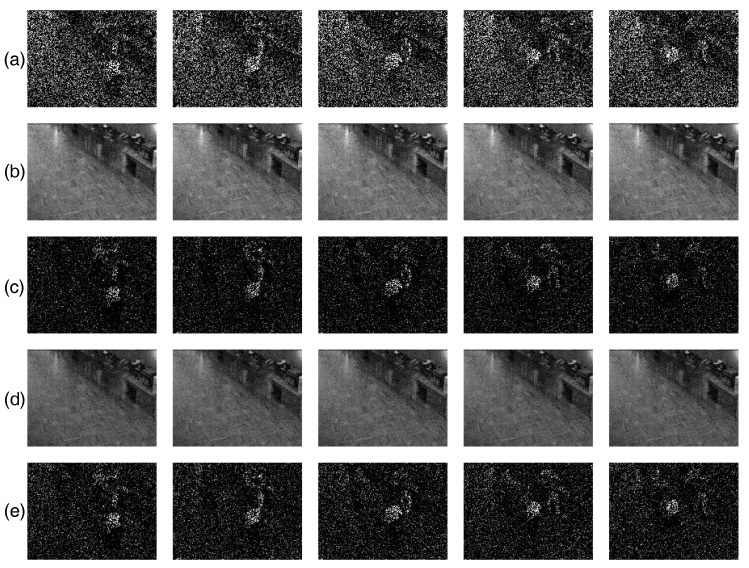
Comparison between RMC-$\ell_{\sigma }$-IHT (Algorithm 1) and RMC-$\ell_{1}$-ADM [17] on extracting the image sequence “Restaurant” with $\rho_{m}=50\%$ and contaminated by two types of non-Gaussian noise with $s_{n}\;=\;0.05$. (**a**) The original image sequence; (**b**) the image sequence with missing entries and contaminated by noise; (**c**) background extracted by RMC-$\ell_{\sigma }$-IHT (Algorithm 1); (**d**) foreground extracted by RMC-$\ell_{\sigma }$-IHT (Algorithm 1); (**e**) background extracted by RMC-$\ell_{1}$-ADM [17]; (**f**) foreground extracted by RMC-$\ell_{1}$-ADM [17].

The running time and relative error are reported in Table 5. From the table, we see that the proposed approach is faster and gives smaller relative errors. To give an intuitive impression, we choose five frames from each image sequence, as shown in Figure 5, from which we can observe that when the image sequences are corrupted by noise ($s_{n}=0.05$) and missing entries, both of the methods can successfully extract the background and foreground images, and it seems that our method performs better because the details of the background images are recovered well, whereas the LAD-based approach does not seem to perform as well as ours where some details of the background are added to the foreground. It can be also observed that none of the two methods can recover the missing entries in the foreground. In order to achieve this, maybe more effective approaches are needed.

## 6. Concluding Remarks

The correntropy loss function has been studied in the literature [20,21] and has found many successful applications [29,30,31]. Learning with correntropy-induced losses could be resistant to non-Gaussian noise and outliers while ensuring good prediction accuracy simultaneously with properly chosen parameter σ. This paper addressed the robust matrix completion problem based on the correntropy loss. The proposed approach was shown to be efficient to deal with non-Gaussian noise and sparse gross errors. The nonconvexity of the proposed approach was due to using the $\ell_{\sigma }$ loss. Based on the above approach, we proposed two nonconvex optimization models and extend them to the more general robust affine rank minimization problems. Two gradient-based iterative schemes to solve the nonconvex optimization problems were offered, with convergence rate results being obtained under proper assumptions. It would be interesting to investigate similar convergence and recoverability results for other redescending-type loss functions-based models. Numerical experiments verified the improved performance of our methods, where empirically, the parameter σ for $\ell_{\sigma }$ is set to 0.5 and λ for the nuclear norm model (6) is $\lambda =\frac{\min\{m,n\}}{10\sqrt{\max\{m,n\}}}$.

## Figures and Tables

**Table 1:** Notations used in the experiments.

Notations	Descriptions
$\rho_{r}$	the ratio of the rank to the dimensionality of a matrix
$\rho_{o}$	the ratio of outliers to the number of entries of a matrix
$\rho_{m}$	the level of missing entries
$s_{n}$	the factor of scale of noise

**Table 2:** Comparison of RMC-$\ell_{\sigma }$-IHT(Algorithm 1), RMC-$\ell_{\sigma }$-IST(Algorithm 3) and RMC-$\ell_{1}$-ADM [17] on CPU time and the relative error on synthetic data. $\rho_{m}=0.3$, $\rho_{r}=0.1$. rel.err, relative error.

$\rho_{m}$	$\rho_{o}$	RMC-$\ell_{\sigma }$-IHT		RMC-$\ell_{\sigma }$-IST		RMC-$\ell_{1}$-ADM	
		Algorithm 1		Algorithm 3		[17]	
		Time	rel.err	Time	rel.err	Time	rel.err
	0.1	15.43	3.80$\times 10^{-03}$	20.53	4.55$\times 10^{-02}$	19.24	**2.58$\times 10^{-06}$**
	0.15	15.31	4.40$\times 10^{-03}$	21.26	4.96$\times 10^{-02}$	18.32	**2.33$\times 10^{-06}$**
	0.2	16.93	5.40$\times 10^{-03}$	22.95	5.53$\times 10^{-02}$	48.97	**2.82$\times 10^{-04}$**
	0.25	19.04	**5.80$\times 10^{-03}$**	26.41	6.23$\times 10^{-02}$	243.80	1.07$\times 10^{-01}$
	0.3	27.10	**7.00$\times 10^{-03}$**	29.47	7.01$\times 10^{-02}$	137.99	3.16$\times 10^{-01}$
0.2	0.35	26.35	**8.00$\times 10^{-03}$**	36.03	8.10$\times 10^{-02}$	99.26	4.86$\times 10^{-01}$
	0.4	23.91	**1.03$\times 10^{-02}$**	37.41	9.41$\times 10^{-02}$	79.85	6.38$\times 10^{-01}$
	0.45	29.64	**1.24$\times 10^{-02}$**	45.68	1.10$\times 10^{-01}$	67.45	7.77$\times 10^{-01}$
	0.5	40.41	**1.69$\times 10^{-02}$**	61.39	1.37$\times 10^{-01}$	60.08	9.52$\times 10^{-01}$
	0.55	60.28	**2.45$\times 10^{-02}$**	103.87	1.80$\times 10^{-01}$	68.52	1.39$\times 10^{+00}$
	0.6	102.19	**3.69$\times 10^{-02}$**	154.04	2.65$\times 10^{-01}$	144.37	2.86$\times 10^{+00}$
	0.1	16.38	5.20$\times 10^{-03}$	24.14	5.66$\times 10^{-02}$	24.81	**2.86$\times 10^{-06}$**
	0.15	20.14	**5.00$\times 10^{-03}$**	23.85	6.41$\times 10^{-02}$	110.67	8.30$\times 10^{-03}$
	0.2	22.83	**6.00$\times 10^{-03}$**	25.92	7.00$\times 10^{-02}$	117.91	1.15$\times 10^{-01}$
	0.25	20.71	**7.00$\times 10^{-03}$**	28.93	7.97$\times 10^{-02}$	118.10	3.08$\times 10^{-01}$
	0.3	20.77	**8.80$\times 10^{-03}$**	32.99	9.21$\times 10^{-02}$	89.56	4.68$\times 10^{-01}$
0.3	0.35	21.28	**8.20$\times 10^{-03}$**	33.72	9.09$\times 10^{-02}$	88.73	4.66$\times 10^{-01}$
	0.4	27.64	**1.15$\times 10^{-02}$**	41.53	1.05$\times 10^{-01}$	75.07	5.98$\times 10^{-01}$
	0.45	32.38	**1.40$\times 10^{-02}$**	48.45	1.23$\times 10^{-01}$	71.14	7.13$\times 10^{-01}$
	0.5	44.53	**1.68$\times 10^{-02}$**	84.67	1.50$\times 10^{-01}$	73.63	8.02$\times 10^{-01}$
	0.55	62.23	**2.26$\times 10^{-02}$**	125.48	1.95$\times 10^{-01}$	78.34	8.84$\times 10^{-01}$
	0.6	92.14	**3.26$\times 10^{-02}$**	241.35	2.78$\times 10^{-01}$	74.09	1.07$\times 10^{+00}$

**Table 3:** Experimental results of RMC-$\ell_{\sigma }$-IST (Algorithm 3), RMC-$\ell_{1}$-ADM [17] and MC-$\ell_{2}$-IST [16] on different images with $\rho_{r}=0.1$, $\rho_{m}=0.3$ and $\rho_{o}$ varying from 0.3 to 0.7.

$\rho_{o}$	Images	Baboon		Camera Man		Lake		Lena		Pepper	
	Method	Time	rel.err	Time	rel.err	Time	rel.err	Time	rel.err	Time	rel.err
	RMC-$\ell_{\sigma }$-IST (Algorithm 3)	**3.17**	1.46$\times 10^{-02}$	**3.55**	**1.74$\times 10^{-02}$**	**3.79**	**1.61$\times 10^{-02}$**	**4.36**	**2.05$\times 10^{-02}$**	**3.80**	**1.10$\times 10^{-02}$**
0.3	RMC-$\ell_{1}$-ADM [17]	32.22	2.86$\times 10^{-02}$	35.87	4.36$\times 10^{-02}$	26.74	4.57$\times 10^{-02}$	20.67	3.98$\times 10^{-02}$	33.08	2.46$\times 10^{-02}$
	MC-$\ell_{2}$-IST [16]	68.33	4.35$\times 10^{+00}$	72.44	4.44$\times 10^{+00}$	68.39	4.14$\times 10^{+00}$	68.68	4.22$\times 10^{+00}$	68.38	3.07$\times 10^{+00}$
	RMC-$\ell_{c}$-IST	5.19	**1.38$\times 10^{-02}$**	5.60	1.83$\times 10^{-02}$	5.24	1.70$\times 10^{-02}$	4.73	2.46$\times 10^{-02}$	4.36	1.61$\times 10^{-02}$
	RMC-$\ell_{\sigma }$-IST (Algorithm 3)	**3.76**	1.73$\times 10^{-02}$	**3.94**	**2.15$\times 10^{-02}$**	**4.69**	**1.96$\times 10^{-02}$**	**4.58**	**2.41$\times 10^{-02}$**	**4.91**	**1.42$\times 10^{-02}$**
0.4	RMC-$\ell_{1}$-ADM [17]	30.93	3.51$\times 10^{-02}$	36.76	5.16$\times 10^{-02}$	26.67	5.48$\times 10^{-02}$	22.41	4.76$\times 10^{-02}$	32.18	3.28$\times 10^{-02}$
	MC-$\ell_{2}$-IST [16]	68.51	5.07$\times 10^{+00}$	68.94	5.08$\times 10^{+00}$	68.09	4.74$\times 10^{+00}$	68.84	4.88$\times 10^{+00}$	68.68	3.54$\times 10^{+00}$
	RMC-$\ell_{c}$-IST	4.88	**1.70$\times 10^{-02}$**	5.73	2.37$\times 10^{-02}$	5.34	2.21$\times 10^{-02}$	5.39	2.89$\times 10^{-02}$	5.56	1.87$\times 10^{-02}$
	RMC-$\ell_{\sigma }$-IST (Algorithm 3)	**4.01**	**2.13$\times 10^{-02}$**	**4.44**	**2.61$\times 10^{-02}$**	**5.29**	**2.40$\times 10^{-02}$**	**5.27**	**2.76$\times 10^{-02}$**	**6.77**	**1.63$\times 10^{-02}$**
0.5	RMC-$\ell_{1}$-ADM [17]	24.95	4.91$\times 10^{-02}$	27.69	6.57$\times 10^{-02}$	22.75	6.92$\times 10^{-02}$	20.74	6.71$\times 10^{-02}$	26.86	3.98$\times 10^{-02}$
	MC-$\ell_{2}$-IST [16]	68.30	5.56$\times 10^{+00}$	69.64	5.62$\times 10^{+00}$	68.71	5.37$\times 10^{+00}$	68.56	5.44$\times 10^{+00}$	68.71	3.91$\times 10^{+00}$
	RMC-$\ell_{c}$-IST	6.63	2.18$\times 10^{-02}$	6.94	2.95$\times 10^{-02}$	5.84	2.90$\times 10^{-02}$	6.10	3.32$\times 10^{-02}$	6.94	2.15$\times 10^{-02}$
	RMC-$\ell_{\sigma }$-IST (Algorithm 3)	**4.98**	**2.65$\times 10^{-02}$**	6.36	**3.37$\times 10^{-02}$**	**7.96**	**3.11$\times 10^{-02}$**	**5.75**	**3.49$\times 10^{-02}$**	**9.52**	**2.20$\times 10^{-02}$**
0.6	RMC-$\ell_{1}$-ADM [17]	15.55	1.41$\times 10^{-01}$	15.21	1.61$\times 10^{-01}$	15.23	1.48$\times 10^{-01}$	15.56	1.38$\times 10^{-01}$	15.95	9.71$\times 10^{-02}$
	MC-$\ell_{2}$-IST [16]	68.22	6.06$\times 10^{+00}$	69.93	6.17$\times 10^{+00}$	68.73	5.77$\times 10^{+00}$	68.34	5.88$\times 10^{+00}$	68.51	4.23$\times 10^{+00}$
	RMC-$\ell_{c}$-IST	7.93	2.70$\times 10^{-02}$	**6.08**	4.51$\times 10^{-02}$	8.19	3.22$\times 10^{-02}$	7.87	3.81$\times 10^{-02}$	10.36	2.85$\times 10^{-02}$
	RMC-$\ell_{\sigma }$-IST (Algorithm 3)	**8.74**	**3.59$\times 10^{-02}$**	**11.37**	**4.41$\times 10^{-02}$**	**11.75**	**4.21$\times 10^{-02}$**	**9.59**	**4.16$\times 10^{-02}$**	**19.95**	**2.69$\times 10^{-02}$**
0.7	RMC-$\ell_{1}$-ADM [17]	44.31	1.90$\times 10^{+00}$	44.63	1.96$\times 10^{+00}$	45.16	1.81$\times 10^{+00}$	43.49	1.85$\times 10^{+00}$	43.88	1.37$\times 10^{+00}$
	MC-$\ell_{2}$-IST [16]	68.54	6.52$\times 10^{+00}$	68.75	6.59$\times 10^{+00}$	69.06	6.18$\times 10^{+00}$	68.41	6.22$\times 10^{+00}$	68.62	4.52$\times 10^{+00}$
	RMC-$\ell_{c}$-IST	13.12	**3.59$\times 10^{-02}$**	23.03	5.03$\times 10^{-02}$	15.19	4.36$\times 10^{-02}$	22.95	4.68$\times 10^{-02}$	14.78	3.86$\times 10^{-02}$

**Table 4:** Experimental results on the image “Building”, contaminated by non-Gaussian noise with varying $\rho_{m}$ and the noise scale.

$s_{n}$	$\rho_{m}$	RMC-$\ell_{\sigma }$-IST		RMC-$\ell_{1}$-ADM		MC-$\ell_{2}$-IST	
		Algorithm 3		[17]		[16]	
		Time	rel.err	Time	rel.err	Time	rel.err
	0.1	0.91	**6.70$\times 10^{-03}$**	2.57	1.76$\times 10^{-02}$	0.59	6.70$\times 10^{-03}$
0.01	0.3	0.90	**9.60$\times 10^{-03}$**	2.40	2.32$\times 10^{-02}$	0.85	9.60$\times 10^{-03}$
	0.5	1.05	**1.44$\times 10^{-02}$**	2.77	3.24$\times 10^{-02}$	1.29	1.44$\times 10^{-02}$
	0.1	1.24	**1.58$\times 10^{-02}$**	1.17	2.16$\times 10^{-02}$	0.82	1.91$\times 10^{-02}$
0.05	0.3	1.11	**2.03$\times 10^{-02}$**	1.37	2.70$\times 10^{-02}$	1.64	3.63$\times 10^{-02}$
	0.5	1.32	**2.49$\times 10^{-02}$**	2.22	3.61$\times 10^{-02}$	1.94	2.88$\times 10^{-02}$
	0.1	2.34	3.31$\times 10^{-02}$	1.08	**3.04$\times 10^{-02}$**	1.35	5.72$\times 10^{-02}$
0.1	0.3	3.30	**3.40$\times 10^{-02}$**	1.44	3.78$\times 10^{-02}$	2.32	4.28$\times 10^{-02}$
	0.5	3.70	**4.66$\times 10^{-02}$**	2.42	5.53$\times 10^{-02}$	3.98	1.55$\times 10^{-01}$

**Table 5:** Experiment results on “Restaurant” contaminated by non-Gaussian noise and 50% missing entries.

$s_{n}$	Method	Time	rel.err
0.01	RMC-$\ell_{\sigma }$-IHT (Algorithm 1)	**70.58**	**9.77$\times 10^{-02}$**
	RMC-$\ell_{1}$-ADM [17]	229.88	1.14$\times 10^{-01}$
0.02	RMC-$\ell_{\sigma }$-IHT (Algorithm 1)	**58.51**	**9.78$\times 10^{-02}$**
	RMC-$\ell_{1}$-ADM [17]	230.24	1.30$\times 10^{-01}$
0.05	RMC-$\ell_{\sigma }$-IHT (Algorithm 1)	**99.87**	**1.14$\times 10^{-01}$**
	RMC-$\ell_{1}$-ADM [17]	221.60	2.37$\times 10^{-01}$

## Appendix A. Lipschitz Continuity of the Gradient of ℓ σ and Some Propositions

The propositions given in the Appendix hold for both $\ell_{\sigma }$ and ${\tilde{\ell }}_{\sigma }$. For simplicity, we only present the formulas for ${\tilde{\ell }}_{\sigma }$. We first give some notations. Let vec(·) be the vectorization operator over any matrix space $R^{s\times t}$, with $\mathrm{vec}\left(B\right)\in R^{st}$ and:

$$\begin{array}{c} \mathrm{vec}{\left(B\right)}_{(i-1)t+j}=B_{ij},\;\;1\leq i\leq s,\;1\leq j\leq t,\;\;\forall B\in R^{s\times t}. \end{array}$$

We further define matrix $A\in R^{p\times mn}$, where:

$$\begin{array}{c} A^{T}=\left[\mathrm{vec}\left(A^{1}\right),\mathrm{vec}\left(A^{2}\right),\ldots ,\mathrm{vec}\left(A^{p}\right)\right]. \end{array}$$

Based on the above notations, the vectorized form of A(X) is written as:

vec(A(X))=Avec(X),

and the gradient of $\ell_{\sigma }$ at *X* can be rewritten as:

$$\begin{array}{c} \mathrm{vec}\left(\nabla \ell_{\sigma }\left(X\right)\right)=A^{T}\Lambda \left(A\mathrm{vec}(X)-b\right), \end{array}$$

where $\Lambda \in R^{p\times p}$ is a diagonal matrix with:

$$\begin{array}{c} \Lambda_{ii}=\exp\left(-{\left(\langle A^{i},X\rangle -b_{i}\right)}^{2}/\sigma^{2}\right),\;\;\;\;1\leq i\leq p. \end{array}$$

Let ${∥A∥}_{2}$ be the spectral norm of *A*. The following proposition shows that the gradient of ${\tilde{\ell }}_{\sigma }$ is Lipschitz continuous.

**Proposition** **A1.** *The gradient of ${\tilde{\ell }}_{\sigma }$ is Lipschitz continuous. That is, for any $X,Y\in R^{m\times n}$, it holds that:*

$$\begin{array}{c} ∥\nabla {\tilde{\ell }}_{\sigma }\left(X\right)-\nabla {\tilde{\ell }}_{\sigma }{\left(Y\right)∥}_{F}\leq {∥A∥}_{2}^{2}{∥X-Y∥}_{F}. \end{array}$$

**Proof.** With notations introduced above, we know that:

$$\begin{array}{ccc} ∥\nabla {\tilde{\ell }}_{\sigma }\left(X\right)-\nabla {\tilde{\ell }}_{\sigma }{\left(Y\right)∥}_{F} & = & ∥A^{T}\Lambda_{X}\left(A\mathrm{vec}(X)-b\right)-A^{T}\Lambda_{Y}\left(A\mathrm{vec}(Y)-b\right){∥}_{F}\\ & \leq & {∥A∥}_{2}{∥\Lambda_{X}\left(A\mathrm{vec}(X)-b\right)-\Lambda_{Y}\left(A\mathrm{vec}(Y)-b\right)∥}_{F}, \end{array}$$

where $\Lambda_{X}$ and $\Lambda_{Y}$ are the diagonal matrices corresponding to $\nabla {\tilde{\ell }}_{\sigma }\left(X\right)$ and $\nabla {\tilde{\ell }}_{\sigma }\left(Y\right)$. It remains to show that:

$$\begin{array}{c} ∥\Lambda_{X}\left(A\mathrm{vec}(X)-b\right)-\Lambda_{Y}\left(A\mathrm{vec}(Y)-b\right){∥}_{F}\leq {∥A∥}_{2}{∥X-Y∥}_{F}. \end{array}$$

By letting $z_{1}=A\mathrm{vec}\left(X\right)-b$ and $z_{2}=A\mathrm{vec}\left(Y\right)-b$, we observe that:

$$\begin{array}{cc} & ∥\Lambda_{X}\left(A\mathrm{vec}(X)-b\right)-\Lambda_{Y}\left(A\mathrm{vec}(Y)-b\right){∥}_{F}^{2}\\ = & \sum_{i=1}^{p}{\left(\exp\left(-z_{1,i}^{2}/\sigma^{2}\right)z_{1,i}-\exp\left(-z_{2,i}^{2}/\sigma^{2}\right)z_{2,i}\right)}^{2}. \end{array}$$

Combining with the fact that for any $t_{1},t_{2}\in R$ and σ>0,

$$\begin{array}{c} |\exp\left(-t_{1}^{2}/\sigma^{2}\right)t_{1}-\exp\left(-t_{2}^{2}/\sigma^{2}\right)t_{2}\left|\leq \right|t_{1}-t_{2}|, \end{array}$$

we have:

$$\begin{array}{cc} & ∥\Lambda_{X}\left(A\mathrm{vec}(X)-b\right)-\Lambda_{Y}\left(A\mathrm{vec}(Y)-b\right){∥}_{F}^{2}\\ \leq & {∥A\mathrm{vec}\left(X\right)-A\mathrm{vec}\left(Y\right)∥}_{F}^{2}\leq {∥A∥}_{2}^{2}{∥X-Y∥}_{F}^{2}. \end{array}$$

As a result, $∥\tilde{\nabla }\ell_{\sigma }\left(X\right)-\tilde{\nabla }\ell_{\sigma }{\left(Y\right)∥}_{F}\leq {∥A∥}_{2}^{2}{∥X-Y∥}_{F}$. This completes the proof. ☐

The following conclusion is a consequence of Proposition A1.

**Proposition** **A2.** *For any $X,Y\in R^{m\times n}$, it holds that:*

(A1) $$\begin{array}{c} {\tilde{\ell }}_{\sigma }\left(X\right)\leq {\tilde{\ell }}_{\sigma }\left(Y\right)+\langle \nabla {\tilde{\ell }}_{\sigma }\left(Y\right),X-Y\rangle +{∥A∥}_{2}^{2}/2{∥X-Y∥}_{F}^{2}. \end{array}$$

**Proposition** **A3.** *Let $\left\{X^{\left(k\right)}\right\}$ be generated by Algorithms 1 or 3 with $\alpha >L={∥A∥}_{2}$. Then, it holds that:*

$$∥X^{\left(k\right)}-X^{\left(k+1\right)}{∥}_{F}\to 0.$$

**Proof.** We first consider $\left\{X^{\left(k\right)}\right\}$ generated by Algorithm 1. Following from the fact that $\mathrm{rank}(X^{\left(k\right)})\leq R$ and $X^{\left(k+1\right)}$ is the best rank-*R* approximation of $Y^{\left(k+1\right)}$, we know that:

$$\begin{array}{cc} & \frac{\alpha }{2}{∥X^{\left(k+1\right)}-X^{\left(k\right)}∥}_{F}^{2}+\langle \nabla {\tilde{\ell }}_{\sigma }\left(X^{\left(k\right)}\right),X^{\left(k+1\right)}-X^{\left(k\right)}\rangle \\ = & \frac{\alpha }{2}{∥X^{\left(k+1\right)}-X^{\left(k\right)}+\frac{1}{\alpha }\nabla \ell_{\sigma }\left(X^{\left(k\right)}\right)∥}_{F}^{2}-\frac{\alpha }{2}{∥\frac{1}{\alpha }\nabla \ell_{\sigma }\left(X^{\left(k\right)}\right)∥}_{F}^{2}\leq 0. \end{array}$$

This together with (A1) gives:

$${\tilde{\ell }}_{\sigma }\left(X^{\left(k+1\right)}\right)\leq {\tilde{\ell }}_{\sigma }\left(X^{\left(k\right)}\right)-\frac{\alpha -L}{2}{∥X^{\left(k+1\right)}-X^{\left(k\right)}∥}_{F}^{2},$$

which implies that the sequence $\left\{{\tilde{\ell }}_{\sigma }\left(X^{\left(k\right)}\right)\right\}$ is monotonically decreasing. Due to the lower boundness of ${\tilde{\ell }}_{\sigma }$, we see that $\lim_{k\to \infty }{∥X^{\left(k+1\right)}-X^{\left(k\right)}∥}_{F}=0$. When $\left\{X^{\left(k\right)}\right\}$ is generated by Algorithm 3, after simple computation, we have that $X^{\left(k+1\right)}$ is the minimizer of:

$$\begin{array}{c} \min_{X}\frac{1}{2}∥X-Y^{\left(k+1\right)}{∥}_{F}^{2}+\frac{\lambda }{\alpha }{∥X∥}_{*}. \end{array}$$

we thus have:

$$\begin{array}{cc} & \frac{\alpha }{2}∥X^{\left(k+1\right)}-X^{\left(k\right)}{∥}_{F}^{2}+\langle \nabla {\tilde{\ell }}_{\sigma }\left(X^{\left(k\right)}\right),X^{\left(k+1\right)}-X^{\left(k\right)}\rangle +\lambda ∥X^{\left(k+1\right)}{∥}_{*}-\lambda {∥X^{\left(k\right)}∥}_{*}\\ = & \frac{\alpha }{2}∥X^{\left(k+1\right)}-Y^{\left(k+1\right)}{∥}_{F}^{2}+\lambda ∥X^{\left(k+1\right)}{∥}_{*}-\frac{\alpha }{2}{∥\frac{1}{\alpha }\nabla \ell_{\sigma }\left(X^{\left(k\right)}\right)∥}_{F}^{2}-\lambda {∥X^{\left(k\right)}∥}_{*}\leq 0. \end{array}$$

This in connection with Proposition A2 reveals:

$${\tilde{\ell }}_{\sigma }\left(X^{\left(k+1\right)}\right)+\lambda ∥X^{\left(k+1\right)}{∥}_{*}\leq {\tilde{\ell }}_{\sigma }\left(X^{\left(k\right)}\right)+\lambda ∥X^{\left(k\right)}{∥}_{*}-\frac{\alpha -L}{2}{∥X^{\left(k+1\right)}-X^{\left(k\right)}∥}_{F}^{2}.$$

Analogously, we have $\lim_{k\to \infty }{∥X^{\left(k+1\right)}-X^{\left(k\right)}∥}_{F}=0$. This completes the proof. ☐
